#  Use of Contrast Echocardiography in Intensive Care and at the Emergency Room

**DOI:** 10.2174/157340311798220467

**Published:** 2011-08

**Authors:** Bernard Cosyns, Bram Roossens, Sophie Hernot, Philippe El Haddad, Hervé Lignian, Luc Pierard, Patrizio Lancellotti

**Affiliations:** 1 UZ Brussel, Cardiology, Free University of Brussels, Belgium; 2 CHIREC, site de Braine, Intensive Care Unit, Free University of Brussels, Belgium; 3 CHU Sart Tilman, Université de Liège, Belgium

**Keywords:** Contrast echocardiography, microbubbles, ultrasound contrast agent, emergency room, intensive care, critically ill.

## Abstract

Bedside echocardiography in emergency room (ER) or in intensive care unit (ICU) is an important tool for managing critically ill patients, to obtain a timely accurate diagnosis and to immediately stratify the risk to the patient’s life.  It may also render invasive monitoring unnecessary. In these patients, contrast echocardiography may improve quality of imaging and also may provide additional information, especially regarding myocardial perfusion in those with suspected coronary artery disease. This article focuses on the principle of contrast echocardiography and the clinical information that can be obtained according to the most frequent presentations in ER and ICU.

## INTRODUCTION

The evaluation and management of patients who arrive at the emergency department (ER) or intensive care unit (ICU) with acute symptoms and/or worrying clinical condition is a daily challenge. Careful history taking-when it is possible-and clinical examination remain the mandatory first steps. It is of importance to obtain a timely accurate diagnosis and to immediately stratify the risk to the patient’s life. Among the possible investigation tools for a precise diagnosis, imaging modalities are frequently required. Echocardiography is the most versatile method. It is non-invasive, can be performed at the bedside, provides rapid results, and avoids exposure of the patient to radiation. Echocardiography is now available in most ER, allowing immediate, standard transthoracic examination. Bedside echocardiography in the ICU is also an important tool in managing critically ill patients, often rendering invasive monitoring unnecessary [1].

In these patients, contrast echocardiography may improve quality of imaging and also may provide additional information, especially regarding myocardial perfusion in those with suspected coronary artery disease. This article focuses on the principle of contrast echocardiography and the clinical information that can be obtained according to the most frequent presentations in ER and ICU.

## ULTRASOUND CONTRAST AGENTS

Contrast on echocardiograms by injecting hand agitated saline solution has been recognized for over 30 years for its ability to opacify vascular structures [2]. The primary mechanism by which injection of such fluids produces ultrasound contrast was determined to be increased backscatter from inclusions of microbubbles (MBB) within the injectant [3]. This way MBB markedly enhanced the blood echo by introducing multiple liquid-gas interfaces. Shortly thereafter, smaller MBB were produced [4] and were rapidly adopted for intracoronary injections in animals and humans [5, 6]. Early attempts to encapsulate the bubbles resulted in agents with improved stability but of a size too large to traverse the pulmonary microvasculature. Therefore, early contrast echocardiography by intravenous injection was used primarily to detect cardiac shunts or examine right heart structures. The first commercial contrast agents of room air, were developed by sonication of 5% human albumin solution, and were small enough to pass through the microcirculation (red blood cell size; i.e. <8 µm). They dissolved rapidly in blood. Consequently their size decreased and they lost their echogenicity. They became commercially available in Europe in 1991 first as Echovist (Berlex, Lachine, QuebecCity, Canada). In 1996, Levovist with galactose microcrystals and with a trace of palmitic acid appeared on the market (Bayer Shering, Berlin Germany). In the U.S. in 1994, Albunex was introduced (Mallinckrodt, St. Louis, Missouri). A second generation contrast agents in which the air in the bubble was changed to higher-molecular-weight gases which resulted in more stable bubbles. Being insoluble in blood, the gas, even when it had escaped from the bubble continued to produce effective ultrasound backscatter by acting as a free gas bubble [7, 8].These new preparations were highly successful in opacifying the left ventricular (LV) cavity and the myocardium from a venous injection. Optison (GE Healthcare, Chalfont StGiles,UK), Definity (BMS, Billerica, Massachusetts) and Sonovue (Bracco, Milan, Italy) are the main representatives of this group. These MBB do not aggregate, are biologically inert and safe [9, 10]. They remain entirely within the vascular space [11], have an intravascular rheology that is very similar to that of erythrocytes [11-13] and are eliminated from the body via the reticulo endothelial system with their gas escaping from the lungs.

## INTERACTION OF CONTRAST AGENTS WITH ULTRASOUND

When ultrasound waves encounter a MBB, it alternately compresses and expands the MBB, depending on the applied acoustic pressure. Therefore the MBB becomes symmetrically larger and smaller in response to the oscillations of the pressure caused by the incident wave. The volume expansion of a MBB is maximal at a specific frequency referred to as the natural resonant frequency and is inversely related to its size [14]. At the resonant frequency the MBB scatters and absorbs ultrasound and can present nonlinear vibrations when the insonifying acoustic pressure is high enough. Consequently, the bubble vibration contains second and higher multiples of the transmitted frequency. Therefore, the backscattered signal from the MBB contains not only the fundamental frequency but also harmonic frequencies, most notably at twice the fundamental frequency (second harmonics). This nonlinear reflection is not shown by tissue, allowing the separation of response from the bubble from that of surrounding tissue. Finally, as the peak pressure becomes more intense, many of these MBB are disrupted, exhibiting an irreversible, transient and intense scattering depending on the type of gas released and its dissolution in the liquid. This scattered signal is also highly nonlinear. The parameter which expressed the energy of the ultrasound beam is the mechanical index (MI). It reflects the approximate exposure to ultrasound pressure at the focus of the beam in an average tissue.

## CONTRAST IMAGING STRATEGIES

The influence of the response of the MBB to ultrasound waves has led to different strategies to visualize the MBB in echo images. These techniques, exploiting the harmonics, have been implemented in commercial clinical ultrasound systems. Very briefly, the main strategies used are divided in two main families: the high power and the low power imaging techniques. The latter are technologies developed to examine the nonlinear responses of MBB. Even low amplitude MBB backscatter can be isolated from tissue signals for processing. This allows continuous low power imaging to be performed, with limited bubble destruction, enabling simultaneous assessment of wall motion and perfusion in real time. Combining high power burst of ultrasound followed by low power imaging, permits to follow the replenishment of the myocardium capillaries with the MBB over time. This approach provides an estimate of myocardial blood volume and of myocardial blood flow [15]. This can also be obtained by using intermittent high power imaging with a progressive increase in pulse interval. 

## CLINICAL APPLICATIONS OF CONTRAST ECHOCARDIOGRAPHY IN THE EMERGENCY ROOM AND INTENSIVE CARE UNIT

### A Global and Regional Systolic Function Evaluation at Rest

The evaluation of left ventricular function is often required for patients at the ER and ICU: to make the diagnosis or to rule out acute coronary syndromes and stratify the risk, in patients with suspicion of acute heart failure, to clarify the etiology of a shock or for hemodynamic assessment of patients with sepsis. 

In patients presenting with chest pain and a suspicion of acute coronary syndrome, echocardiography plays a key role for the diagnosis, the risk stratification and the management of the disease. Patients with acute chest pain account for a notable proportion (20–30%) of medical admissions to the emergency department [16]. A vast majority of patients have atypical chest pain and/or normal or nondiagnostic ECG; early determination of serum troponin frequently is negative. Marginal elevations of troponin in this clinical setting result in uncertainty. The primary role of rest echocardiography when performed in the emergency room is to assess the presence and extent of regional wall motion abnormalities. The absence of dyssynergy cannot definitely exclude a recent episode of ischemia. Moreover, the presence of a dyssynergic segment may be difficult to interprete (ongoing ischemia or old myocardial infarction) and can be present in other pathologic conditions than ischemic disease (myocarditis, right ventricular overload, left bundle branch block, pacemaker) but the main limitation is that the recording of reliable images can be technically difficult if a skilled echocardiographer is not available. The infusion of contrast agent opacifying the left ventricle results in improvement of the endocardial border detection and has been shown to improve the sensitivity and specificity of echocardiography at rest and during stress for the diagnosis of acute coronary syndromes [17-23]. In this setting, contrast echocardiography simultaneously allows the observation of myocardial perfusion, providing incremental information. For a given regional function, normal perfusion was indicative of a very low risk whereas abnormal perfusion identified a high risk for acute coronary syndrome [24]. Patients with normal perfusion and function have excellent outcome for early events, whereas those in whom both are abnormal have the worst outcome. Intermediate outcome is noted in those with normal perfusion despite abnormal function [25-30]. 

Once the diagnosis of acute coronary syndrome is established, contrast echocardiography improves the detection of complications and a better risk stratification of these patients. Considering the critical issue of left ventricular rupture (LVR) with pseudoaneurysm formation after ST-elevation acute myocardial infarction (AMI), there are approximately 500,000 ST-elevation AMIs in the U.S. annually, of which 1% to 6% involve LVR [31]. Free wall rupture may result in pseudoaneurysm, whereby the extravasation of blood into the pericardial space is prevented by adherence of the parietal pericardium to the underlying epicardium. A prompt surgical correction is always indicated for pseudoaneurysm to prevent rupture [32]. The sensitivity of transthoracic echocardiography for the diagnosis of left ventricular (LV) pseudoaneurysm is only 26%, often because of inadequate imaging windows or failure to obtain a good tomographic view [33]. However, the appearance of an intravenous contrast agent in the pericardial space is not dependent on tomographic slices and is diagnostic of LV pseudoaneurysm [34]. In a study by García-Fernández *et al*., ultrasound contrast agents were used for the diagnosis of LV pseudoaneurysm in 19 cases. In thirteen of them, contrast was required to make the diagnosis whereas in other 4 patients, the diagnosis was suspected by noncontrast echocardiography but confirmed by contrast administration. In the two remaing patients, suspected LV pseudoaneurysm was ruled out by contrast echocardiography, thereby preventing unnecessary emergent operations [35]. On the other hand, ultrasound contrast agents (UCA) frequently result in the diagnosis of LV apical thrombus in AMI, which is a major risk factor for death or stroke [36].

Numerous patients, especially the elderly, are admitted to the emergency room for acute decompensated heart failure. Acute cardiogenic pulmonary oedema may result from acute events or from acute deterioration of a chronic disease. The main mechanisms include reduced outflow, reduced inflow or backward flow. Rapid distinction between heart failure due to systolic versus diastolic dysfunction should be obtained since there are significant differences in treatment. Therefore, the evaluation of systolic performance is critical and a good visualisation of the endocardium improves confidence of the operator in these often difficult to image patients. Patients in the intensive care unit also remain in technically challenging to obtain adequate echocardiographic images. The frequent use of mechanical ventilation, presence of chest bandages, and difficulty in positioning the patient, poor lighting conditions are factors that impair image quality. Contrast echocardiography may play an important role in this setting [37]. 

On the other hand, the precise evaluation of aortic stenosis severity is mandatory and contrast may help to enhance Doppler signal. Finally, in patients with acute heart failure and no previous history of coronary artery disease, myocardial perfusion echocardiography is able to determine the presence coronary artery disease, and so, is able to identify viable myocardium at the bedside without requiring a more cumbersome assessment through SPECT, PET, or magnetic resonance imaging (MRI) [38].

### B Regional Systolic Function During Stress

Exercise echocardiography and exercise SPECT have been shown to provide comparable short term prognostic information in the triage of chest pain patients, allowing safe early discharge; the negative predictive value was 97% in both methods, but exercise echocardiography is preferable because of a higher positive predictive value [39]. Pharmacological stress echocardiography can be used in patients unsuitable for exercise testing. Graded dobutamine infusion with addition of atropine if necessary, or high dose dipyridamole and atropine, can be used as stressors. Early dobutamine stress after admission to the emergency room has been shown to be feasible and safe. Pre-discharge dobutamine stress echocardiography has important and independent prognostic value in low risk, troponin negative, chest pain patients [40]. As compared to exercise ECG, dobutamine stress echocardiography was found to be more cost effective: the mean length of stay in the hospital was lower; and no event occurred in a 2 month follow-up in patients with a normal dobutamine test, whereas the event rate was 11% in patients with normal exercise ECG [41]. Reduced endocardial border definition is exacerbated during stress because of chest wall motion during hyperventilation and cardiac translational movement during tachycardia. With fundamental imaging, inadequate endocardial definition has been reported in up to 30% of stress echos. In addition, Hoffman *et al* demonstrated that suboptimal studies have worse reproducibility and a poorer inter-observer variability, with inter-institutional institutional observer agreement as low as 43% for studies with poor image quality [20]. Contrast enhanced echocardiography has proven to increase the accuracy and reproducibility of regional wall motion assessment during stress [17, 18]. 

Contrast echocardiography for enhancing LV borders in suboptimal studies actually represents the main indication of ultrasound contrast agents and is especially useful in approximately 10% to 20% of routine echocardiographic examinations. During dobutamine echocardiography, the sensitivity of contrast myocardial perfusion echocardiography has also been shown to be higher than that of wall motion at both maximal and intermediate doses of dobutamine for the detection of coronary artery disease and in predicting events [42-46].

### C Tissue Characterization

**Fig. (1):**
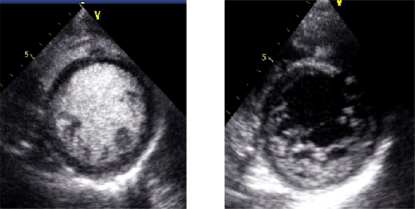
Parasternal short axis view with and without contrast in a patient presenting with acute heart failure. The contrast injection allows a better separation of the compacted (yellow dots) and non-compacted layer and to apply the criteria of diagnosis in isolated non-compaction of the left ventricle.

Contrast echocardiography also has been of value in the structural assessment of the left and right ventricles, the atria, and the great vessels. Ultrasound contrast agents play a key role in the definition of left ventricular apical abnormalities, in complications of myocardial infarction, and in cases of intracardiac masses [47-51]. It may also help to characterize the myocardium in patients with heart failure as illustrated here to allow the distinction between non-compacted and compacted layer in a patient with heart failure and severe left ventricular dysfunction and with non-compaction of the left ventricle (Fig. **1**). More recently, in patients with Tako-Tsubo, the potential use of contrast imaging to better define the variance of typical left ventricular ballooning finding in this condition has been emphasized [52] and on the other hand myocardial perfusion imaging has also been shown to be useful [53-55].

### D Doppler Signal Enhancement

**Fig. (2):**
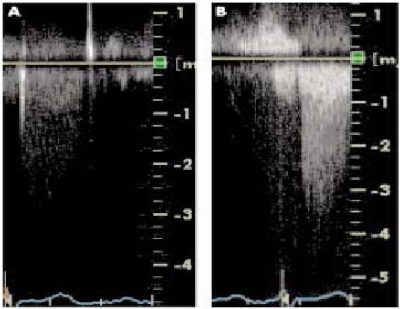
Aortic stenosis flow Doppler tracing (**A**) without and (**B**) with contrast enhancement.

Doppler echocardiographic assessment of blood flow velocities in the heart and the great vessels is a standard part of the cardiac ultrasound examination. Contrast enhancement of the Doppler signal has been shown to be of value when the signal is weak or technically suboptimal. Velocity measurement in patients suspected of aortic stenosis may be enhanced with echocardiographic contrast agents as shown in Fig. **2** [56].

### E Pericardiocentesis

**Fig. (3):**
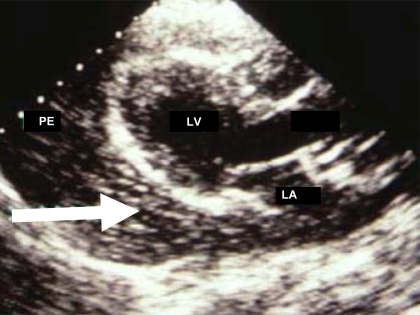
Pericardial effusion (PE) during perocardiocentesis and injection of microbubbles (Arrow) into the needle used for the puncture. La indicates left atrium and LV left ventricle.

Echocardiography plays a major role in pericardiocentesis to determine the distribution and the depth of the effusion. During the procedure, continuous echocardiography has been proposed, eventually combined to the injection of agitated physiologic serum to generate contrast and define the tip of the needle (Fig. **3**).

## SAFETY ISSUE OF CONTRAST ECHOCARDIOGRAPHY IN THE CRITICALLY ILL

Although ultrasound contrast agents have proven utility in the diagnosis and management of critically ill patients [37, 57-59], concern persists regarding the safety of these compounds, particularly in these patients. Recently published single center data demonstrated no increased mortality in hospitalized patients undergoing echocardiography with ultrasound contrast agents in comparison with patients undergoing noncontrast- enhanced examinations [60]. These findings were recently corroborated in large multicenter cohorts [61, 62]. Additionally, multivariate logistic regression modelling demonstrated a significantly lower risk of mortality in the UCA group compared with the no contrast group (24% decreased risk), a finding that may be surprising given recent safety concerns [61]. In a recent prospective study performed early after acute myocardial infarction, administration of echo contrast did not induce any significant change in vital signs, physical examination, and ECG. There were no serious adverse events, and minor events occurred only in five patients [63]. More recently, a study over 22,000 patients who received ultrasound contrast agents, nearly 3000 of whom had critical illness. No association was found between contrast use and the same day mortality [64]. Therefore, in most of these circumstances, the benefit of ultrasound contrast agents far outweighs their risks.

## CONCLUSIONS

In the acutely ill patients, transthoracic image quality is often a problem and the use of ultrasound contrast agents to enhance left ventricular opacification improves the accuracy of echocardiography by rendering examination interpretable. Consequently, this may have implications in patient management. Moreover, perfusion study with contrast echocardiography may add information for diagnosis and risk stratification in patients presenting at the emergency room or hospitalized in intensive care unit. Although there were several concerns regarding the safety with the use of ultrasound contrast agents in the acutely ill patient, recent studies have provided reassurance about the use of contrast in this setting.

## References

[1] McLean AS, Needham A, Stewart D, Parkin R (1997). Estimation of cardiac output by noninvasive echocardiographic techniques in the critically ill subject. Anaesth Intensive Care.

[2] Gramiak R, Shah PM (1968). Echocardiography of the aortic root. Invest Radiol.

[3] Almeida Mbbs AA, Thomson Fracp HL, Burstow Fracp DJ, Tam Fracs RKW (1998). Transesophageal Echocardiography in an Operation for Pulmonary Arteriovenous Malformation. Ann Thoracic Surg.

[4] Feinstein SB, Ten Cate FJ, Zwehl W (1984). Two-dimensional contrast echocardiography. I. *In vitro* development and quantitative analysis of echo contrast agents. J Am Coll Cardiol.

[5] Keller MW, Glasheen W, Smucker ML, Burwell LR, Watson DD, Kaul S (1988). Myocardial contrast echocardiography in humans. II. Assessment of coronary blood flow reserve. J Am Coll Cardiol.

[6] Keller MW, Glasheen W, Teja K, Gear A, Kaul S (1988). Myocardial contrast echocardiography without significant hemody-namic effects or reactive hyperemia: a major advantage in the imaging of regional myocardial perfusion. J Am Coll Cardiol.

[7] Klibanov AL, Rasche PT, Hughes MS (2002). Detection of Individual Microbubbles of an Ultrasound Contrast Agent: Fundamental and Pulse Inversion Imaging. Academic Radiology.

[8] Postema M, van Wamel A, ten Cate FJ, de Jong N (2005). High-speed photography during ultrasound illustrates potential therapeutic applications of microbubbles. Med Phys.

[9] Lindner JR, Ismail S, Spotnitz WD, Skyba DM, Jayaweera AR, Kaul S (1998). Basic Science Reports - Albumin Microbubble Persistence During Myocardial Contrast Echocardiography Is Associated With Microvascular Endothelial Glycocalyx Damage. Circulation - Hagertown.

[10] Skyba DM, Camarano G, Goodman NC, Price RJ, Skalak TC, Kaul S (1996). Hemodynamic characteristics, myocardial kinetics and microvascular rheology of FS-069, a second-generation echocardiographic contrast agent capable of producing myocardial opacification from a venous injection. J Am Coll Cardiol.

[11] Lindner JR, Song J, Jayaweera AR, Sklenar J, Kaul S (2002). Microvascular rheology of Definity microbubbles after intraarterial and intravenous administration. Journal of the American Society of Echocardiography.

[12] Jayaweera AR, Edwards N, Glasheen WP, Villanueva FS, Abbott RD, Kaul S (1994). *In Vivo* Myocardial Kinetics of Air-Filled Albumin Microbubbles During Myocardial Contrast Echocardiography: Comparison With Radiolabeled Red Blood Cells. Circulation Research.

[13] Keller MW, Segal SS, Kaul S, Duling B (1989). The behavior of sonicated albumin microbubbles within the microcirculation: a basis for their use during myocardial contrast echocardiography. Circ Res.

[14] Medwin H (1977). Counting bubbles acoustically: a review. Ultrasonics.

[15] Wei K, Jayaweera AR, Firoozan S, Linka A, Skyba DM, Kaul S (1998). Quantification of myocardial blood flow with ultrasound-induced destruction of microbubbles administered as a constant venous infusion. Circulation.

[16] Blatchford O, Capewell S, Murray S, Blatchford M (1999). Emergency medical admissions in Glasgow: general practices vary despite adjustment for age, sex, and deprivation. Br J Gen Pract.

[17] Cosyns B, Lancellotti P, Van Camp G, Droogmans S, Schoors D (2008). Head to head comparison of transesophageal and transthoracic contrast-enhanced echocardiography during dobutamine administration for the detection of coronary artery dis-ease. Int J Cardiol.

[18] Dolan MS, Riad K, El-Shafei A (2001). Effect of intravenous contrast for left ventricular opacification and border definition on sensitivity and specificity of dobutamine stress echocardiography compared with coronary angiography in technically difficult patients. Am Heart J.

[19] Hoffmann R, Borges A, Kasprzak J (2004). Contrast-enhanced echocardiography improves agreement on the assessment of ejection fraction and left ventricular volumes: A multicenter study. J Am Coll Cardiol.

[20] Hoffmann R, von Bardeleben S, ten Cate F (2005). Assessment of Systolic Left Ventricular Function: A Multi-centre Comparison of Cineventriculography, Cardiac Magnetic Resonance Imaging, Unenhanced and Contrast-enhanced Echocardiography. ACC Curr J Rev - Am Coll Cardiol.

[21] Hundley WG, Kizilb+0.ash AM, Afridi I, Franco F, Peshock RM, Grayburn PA (1999). Effect of contrast enhancement on transthoracic echocardiographic assessment of left ventricular regional wall motion. Am J Cardiol.

[22] Malm S, Frigstad S, Sagberg E, Larsson H, Skjaerpe T (2004). Accurate and reproducible measurement of left ventricular volume and ejection fraction by contrast echocardiography: a comparison with magnetic resonance imaging. J Am Coll Cardiol.

[23] Thomson HL, Basmadjian A-J, Rainbird AJ (2001). Contrast echocardiography improves the accuracy and reproducibility of left ventricular remodeling measurements - A prospective, randomly assigned, blinded study. Journal of the Am Coll Cardiol.

[24] Tong KL, Kaul S, Wang XQ (2005). Myocardial contrast echocardiography versus Thrombolysis In Myocardial Infarction score in patients presenting to the emergency department with chest pain and a nondiagnostic electrocardiogram. J Am Coll Cardiol.

[25] Kaul S, Senior R, Firschke C (2004). Incremental value of cardiac imaging in patients presenting to the emergency department with chest pain and without ST-segment elevation: a multicenter study. Am Heart J.

[26] Korosoglou G, Labadze N, Giannitsis E (2005). Usefulness of real-time myocardial perfusion imaging to evaluate tissue level reperfusion in patients with non-ST-elevation myocardial infarction. Am J Cardiol.

[27] Rinkevich D, Kaul S, Wang XQ (2005). Regional left ventricular perfusion and function in patients presenting to the emergency department with chest pain and no ST-segment elevation. Eur Heart J.

[28] Senior R, Ashrafian H (2005). Detecting acute coronary syndrome in the emergency department: the answer is in seeing the heart: why look further?. Eur Heart J.

[29] Tong KL, Kaul S, Wang X-Q (2005). Myocardial Contrast Echocardiography Versus Thrombolysis in Myocardial Infarction Score in Patients Presenting to the Emergency Department With Chest Pain and a Nondiagnostic Electrocardiogram. J Am Coll Cardiol.

[30] Vannan MA, Narula J (2005). Ischemic versus nonischemic chest pain in the emergency room: echoes of contrast. J Am Coll Cardiol.

[31] Antman EM, Anbe DT, Armstrong PW (2004). ACC/AHA guidelines for the management of patients with ST-elevation myocardial infarction, A report of the American College of Cardiology/American Heart Association Task Force on Practice Guidelines (Committee to Revise the 1999 Guidelines for the Management of patients with acute myocardial infarction). J Am Coll Cardiol.

[32] Main ML, Goldman JH, Grayburn PA (2007). Thinking Outside the "Box"--The Ultrasound Contrast Controversy. J Am Coll Cardiol.

[33] Frances C, Romero A, Grady D (1998). Left ventricular pseudoaneurysm. J Am Coll Cardiol.

[34] Uno K, Takenaka K, Asada K (2006). Diagnosis of subacute cardiac rupture by contrast echocardiography. J Am Soc Echocardiogr.

[35] Garcia-Fernandez MA, Macchioli RO, Moreno PM (2001). Use of contrast echocardiography in the diagnosis of subacute myocardial rupture after myocardial infarction. J Am Soc Echocardiogr.

[36] Mansencal N, Nasr IA, Pilliere R (2007). Usefulness of contrast echocardiography for assessment of left ventricular thrombus after acute myocardial infarction. Am J Cardiol.

[37] Cosyns B, El Haddad P, Lignian H (2004). Contrast harmonic imaging improves the evaluation of left ventricular function in ventilated patients: comparison with transesophageal echocardiography. Eur J Echocardiogr.

[38] Senior R, Janardhanan R, Jeetley P, Burden L (2005). Myocardial Contrast Echocardiography for Distinguishing Ischemic From Nonischemic First-Onset Acute Heart Failure: Insights Into the Mechanism of Acute Heart Failure. Circulation - Hagertown.

[39] Conti A, Sammicheli L, Gallini C, Costanzo EN, Antoniucci D, Barletta G (2005). Assessment of patients with low-risk chest pain in the emergency department: Head-to-head comparison of exercise stress echocardiography and exercise myocardial SPECT. Am Heart J.

[40] Bholasingh R, Cornel JH, Kamp O (2003). Prognostic value of predischarge dobutamine stress echocardiography in chest pain patients with a negative cardiac troponin T. J Am Coll Cardiol.

[41] Nucifora G, Badano LP, Sarraf-Zadegan N (2007). Comparison of early dobutamine stress echocardiography and exercise electrocardiographic testing for management of patients presenting to the emergency department with chest pain. Am J Cardiol.

[42] Elhendy A, O'Leary EL, Xie F, McGrain AC, Anderson JR, Porter TR (2004). Comparative accuracy of real-time myocardial contrast perfusion imaging and wall motion analysis during dobutamine stress echocardiography for the diagnosis of coronary artery disease. J Am Coll Cardiol.

[43] Elhendy A, Porter TR (2005). Assessment of myocardial perfusion with real-time myocardial contrast echocardiography: Methodology and clinical applications. J Nuclear Cardiol.

[44] Moir S, Haluska BA, Jenkins C, Fathi R, Marwick TH (2005). Incremental benefit of myocardial contrast to combined dipyridamole-exercise stress echocardiography for the assessment of coronary artery disease. ACC Curr J Rev.

[45] Tsutsui JM, Elhendy A, Anderson JR, Xie F, McGrain AC, Porter TR (2005). Prognostic value of dobutamine stress myocardial contrast perfusion echocardiography. Circulation.

[46] Tsutsui JM, Xie F, Cloutier D, Kalvaitis S, Elhendy A, Porter TR (2008). Real-time dobutamine stress myocardial perfusion echocardiography predicts outcome in the elderly. Eur Heart J.

[47] Stratton JR, Lighty GW, Pearlman AS, Ritchie JL (1982). Detection of left ventricular thrombus by two-dimensional echo-cardiography: sensitivity, specificity, and causes of uncertainty. Circulation.

[48] Moreno R, Zamorano JL, Almeria C (2002). Usefulness of contrast agents in the diagnosis of left ventricular pseudoaneurysm after acute myocardial infarction. Eur J Echocardiogr.

[49] Zhu H, Muro T, Hozumi T (2002). [Usefulness of left ventricular opacification with intravenous contrast echocardiography in patients with asymptomatic negative T waves on electrocardiography]. J Cardiol.

[50] Andresen H, Kaag N, Potratz J (2005). Non-compaction of ventricular myocardium and contrast-enhanced echocardiography. Z Kardiol.

[51] Kirkpatrick JN, Wong T, Bednarz JE (2004). Differential diagnosis of cardiac masses using contrast echocardiographic perfusion imaging. J Am Coll Cardiol.

[52] Hurst RT, Prasad A, Askew JW, Sengupta PP, Tajik AJ (2010). Takotsubo cardiomyopathy: a unique cardiomyopathy with variable ventricular morphology. JACC Cardiovasc Imaging.

[53] Galiuto L, De Caterina AR, Porfidia A (2010). Reversible coronary microvascular dysfunction: a common pathogenetic mechanism in Apical Ballooning or Tako-Tsubo Syndrome. Eur Heart J.

[54] Galiuto L, Gabrielli FA, Lanza GA, Porfidia A, Paraggio L, Barchetta S (2010). Influence of left ventricular hypertrophy on microvascular dysfunction and left ventricular remodelling after acute myocardial infarction. Eur J Echocardiogr.

[55] Galiuto L, Paraggio L, Liuzzo G, de Caterina AR, Crea F (2010). Predicting the no-reflow phenomenon following successful percutaneous coronary intervention. Biomark Med.

[56] Nakatani S, Imanishi T, Terasawa A, Beppu S, Nagata S, Miyatake K (1992). Clinical application of transpulmonary contrast-enhanced Doppler technique in the assessment of severity of aortic stenosis. J Am Coll Cardiol.

[57] Kornbluth M, Liang DH, Brown P, Gessford E, Schnittger I (2000). Clinical Investigations - Imaging/Diagnostic Testing - Contrast echocardiography is superior to tissue harmonics for assessment of left ventricular function in mechanically ventilated patients. Am Heart J.

[58] Reilly JP, Tunick PA, Timmermans RJ, Stein B, Rosenzweig BP, Kronzon I (2000). Contrast echocardiography clarifies uninterpretable wall motion in intensive care unit patients. J Am Coll Cardiol.

[59] Yong Y, Wu D, Fernandes V (2002). Diagnostic accuracy and cost-effectiveness of contrast echocardiography on evaluation of cardiac function in technically very difficult patients in the intensive care unit. Am J Cardiol.

[60] Kusnetzky LL, Khalid A, Khumri TM, Moe TG, Jones PG, Main ML (2008). Acute Mortality in Hospitalized Patients Undergoing Echocardiography With and Without an Ultrasound Contrast Agent: Results in 18,671 Consecutive Studies. J Am Coll Cardiol.

[61] Main ML, Ryan AC, Davis TE, Albano MP, Kusnetzky LL, Hibberd M (2008). Acute mortality in hospitalized patients undergoing echocardiography with and without an ultrasound contrast agent (multicenter registry results in 4,300,966 consecutive patients). Am J Cardiol.

[62] Wei K, Mulvagh SL, Carson L (2008). The Safety of Definity and Optison for Ultrasound Image Enhancement: A Retrospective Analysis of 78,383 Administered Contrast Doses. J Am Soc Echocardiogr.

[63] Nucifora G, Marsan NA, Siebelink HM (2008). Safety of contrast-enhanced echocardiography within 24 h after acute myocardial infarction. Eur J Echocardiogr.

[64] Exuzides A, Main ML, Colby C, Grayburn PA, Feinstein SB, Goldman JH (2010). A retrospective comparison of mortality in critically ill hospitalized patients undergoing echocardiography with and without an ultrasound contrast agent. JACC Cardiovasc Imaging.

